# Reliability and Validity Study of the Chamorro Assisted Gait Scale for People with Sprained Ankles, Walking with Forearm Crutches

**DOI:** 10.1371/journal.pone.0155225

**Published:** 2016-05-11

**Authors:** Gema Chamorro-Moriana, Carmen Ridao-Fernández, Joaquín Ojeda, Marisa Benítez-Lugo, José Luis Sevillano

**Affiliations:** 1 Department of Physiotherapy, Research Group “Area of Physiotherapy CTS-305,” University of Seville, Seville, Spain; 2 Department of Mechanics and Manufacture, Research Group “Mechanical Engineering,” University of Seville, Seville, Spain; 3 Department of Architecture and Technology of Computers, Research Group “Robotics and Technology of Computers,” University of Seville, Seville, Spain; Fraunhofer Research Institution of Marine Biotechnology, GERMANY

## Abstract

**Objective:**

The aim of this study was to design and validate a functional assessment scale for assisted gait with forearm crutches (Chamorro Assisted Gait Scale—CHAGS) and to assess its reliability in people with sprained ankles.

**Design:**

Thirty subjects who suffered from sprained ankle (anterior talofibular ligament first and second degree) were included in the study. A modified Delphi technique was used to obtain the content validity. The selected items were: pelvic and scapular girdle dissociation(1), deviation of Center of Gravity(2), crutch inclination(3), steps rhythm(4), symmetry of step length(5), cross support(6), simultaneous support of foot and crutch(7), forearm off(8), facing forward(9) and fluency(10). Two raters twice visualized the gait of the sample subjects which were recorded. The criterion-related validity was determined by correlation between CHAGS and Coding of eight criteria of qualitative gait analysis (Viel Coding). Internal consistency and inter and intra-rater reliability were also tested.

**Results:**

CHAGS obtained a high and negative correlation with Viel Coding. We obtained a good internal consistency and the intra-class correlation coefficients oscillated between 0.97 and 0.99, while the minimal detectable changes were acceptable.

**Conclusion:**

CHAGS scale is a valid and reliable tool for assessing assisted gait with crutches in people with sprained ankles to perform partial relief of lower limbs.

## Introduction

Human gait is one of the main functions of human beings[1] which has led to gait re-education becoming an increasingly important part of physical therapy treatments.

The need for functional gait evaluations involves the creation of a variety of assessment mechanisms[1,2], such as force or pressure plates, 3D motion analysis or observational gait scales amongst others. Systems based on observational analysis have become an interesting option due to their ease-of-use and low cost. Besides, they are feasible for daily practice[3,4] as well as in the research field[5]. Thus, scales and questionnaires are frequently used effectively among health professionals to describe gait alterations and their evolution during re-education[1].

These tools allow therapists and patients to work at a high degree of objectivity to quantify functional assessment which objectifies progressions in the applied treatments[6]. Furthermore, gait assessment scales are necessary to reach accurate decisions, unify the language of professionals and formalize treatments[2], thereby achieving their performance objectively, comparing results and progressing scientifically in our field[7,8].

Currently, the existing specific functional gait measures are aimed at patients with neurological, geriatric and cardiovascular diseases. Amongst these tools are: Tinetti Scale to assess gait and balance[5] which contains adaptations for neurological patients and elderly people; Gait and balance[1] and Parkinson Dynamic Gait Scale[9], amongst others, intended for Parkinson patients[10]; Wisconsin Gait Scale useful in hemiplegia[11]; Observational Gait Scale for children with cerebral palsy[3]; Ten-Meter Walk Test, used in strokes and other neurological disorders[12]; or Six-Minute Walk Test[13], designed for patients with cardiovascular diseases. However, there are many patients who do not have these pathologies, yet require assessment for abnormal gait over a long time due to a lower limb musculoskeletal injury[14]. None of the mentioned scales (neurologic, geriatric and cardiovascular) are used to evaluate this kind of lesion. Besides, there are no specific scales for musculoskeletal injuries nor assisted gait measurement, although this is a modality employed by many patients during their functional recovery. A sprained ankle, which is one of the most frequent pathologies that requires the use of crutches, involves a weight relief due to inflammation and swelling, in addition to the ligament not being able to bear tension[15].

Assisted gait alterations cause corporal misalignments, which could produce muscular disorders (for example, muscle fatigue of quadratus lumborum), articular overloads, prepatellar bursitis, among other things[16]. In addition to these, longer time treatments and relapses are due to these alterations. To prevent and correct gait disorders it is necessary to quantify not only loads[17] but also the rest of gait parameters[18] such as the deviation of the Center of Gravity (COG), forearm crutch inclination and simultaneity of foot support and crutch. These alterations are directly related to the coordinative skills and level of learning of the patients, since assisted gait is considered a dual task[19]. Therefore, the alterations cited depend on the use of the crutch and not on the pathology itself. Due to this, the aim of this study was to design and assess the reliability and validity of the Chamorro Assisted Gait Scale (CHAGS) in people with sprained ankles which is a new means for evaluating aided gait with forearm crutches to partially relieve an affected member due to a musculoskeletal injury whether surgically intervened or not.

## Methods

### Ethics statement

The subjects were informed about the study both orally and written, after which they signed a consent form in accordance with the Helsinki Declaration. This study was approved by the Ethics Committee of the Hospital Universitario Virgen Macarena, Seville, Spain.

### Participants

The sample was selected in a non-probabilistic and convenience sampling. A standardized error factor of 0.36 with a confidence level of 95% was used. Therefore, the sample consisted of 30 subjects (14 men and 16 women), who suffered from sprained ankle ligaments. The mean age was 34.27 years, SD = 11.24 with a minimum of 18 and a maximum of 60.

The inclusion criteria were: aged between 18 and 60 years old; clinical diagnosis of sprained ankle (anterior talofibular ligament first and second degree); requirement of a partial discharge of the affected limb; a learning process for the correct use of crutches during their functional recovery; ability to be in a treatment phase where they were allowed to load 90% of their body weight on the affected member (last progression in partial discharges before achieving a normal gait)[20,21]; skill to perform assisted gait with free cadence for two minutes non-stop without pain, (according to their physiotherapist’s recommendations); and ability to pass a simple static equilibrium test which consisted of maintaining monopodal balance for 30 seconds on the healthy foot without suffering any great bodily movements[22].

The exclusion criteria were: an evident general coordination and physical ability disorder which could alter the normal or assisted gait.

### Procedure

The trials were videotaped under laboratory conditions (with the same artificial light and temperature) on an 8 meter walkway. The cameras were situated in frontal and lateral positions.

After a period of familiarization with the specific study (walkway, researchers, camera, etc.) the participants wore short, tight clothes when doing the route with different modalities of assisted gait with crutches to perform a partial load of a lower limb: with a contra-lateral crutch (unilateral) and both crutches (bilateral). The gait in each of the modalities lasted two minutes. Walking was at free cadence and there was simultaneous support for heel and crutch at two points[23]. The height of the chosen crutch is the one that produces an elbow flexion of 20° to 30°[24]. The force applied was chosen by the subject in order to achieve heterogeneous load amounts, provided that it did not distort the correct technical gesture of the gait. Said loads were monitored by the Gema Chamorro System (GCH System), which measures the loads exerted on the forearm crutches during aided gait[25].

### Development of the CHAGS

The systematic development processes of the CHAGS scale followed previous guidelines and recommendations[26,27].

The preliminary version of the CHAGS was developed based on the analyses of two different sources information.

*1*^*st*^.*- Published evidence*: A comprehensive and critical literature review was carried out to identify the variables of interest and to guide the selection of item format and characteristics as well as the creation of the scoring model. The chosen scales were Tinetti Scale[5], Clinical Gait and Balance Scale[1], Dynamic Parkinson Gait Scale[9], Coding of eight criteria of qualitative gait analysis or “Codification des huit critères d´analyse qualitative de la marche” by Viel (Viel Coding)[28], Rivermead Visual Gait Assessment form[29] and Shaw Gait Assessment Tool[30]. The items extracted were: step symmetry, step length, step duration, step continuity and rhythmicity, trunk deviations, synchrony between arms and legs, attitude during gait, gait variability and walking while performing a cognitive dual-task. *2*^*nd*^.*- Clinical observation*: New items had to be added by means of clinical observation as there were specific parameters of aided gait with crutches. This observation was made in healthy subjects, in addition to subjects with pathology, so we could confirm that the selected parameters depended on the use of the crutch and not on the pathology itself. In order to determine content validity of the tool, the selected variables were proposed to five experts in gait assessment and gait training. Experts used a modified Delphi technique [31] to set the ten-item version of the CHAGS. The final version consisted of a five-point ordinal scoring scale ranging from 0 to 4. A high score reflected a correct assisted gait (maximum score: 40 for unilateral and 36 for bilateral). In any case, scale interpretation is not achieved globally but item-by-item. Therefore, a result of 4 in each item indicates a correct gait, a score of 3 is acceptable and a result ≤2 corresponds to a non-acceptable gait. The completed Scale and the description of each score are presented in S1 Appendix.

The items were:

-Pelvic and scapular girdle dissociation (PSGD).
-Deviation from the COG.
-Crutch inclination.
-Step rhythm.
-Symmetry of step length.
-Cross support.
-Simultaneous support of foot and crutch.
-Forearm off.
-Facing forward.
-Fluency.

This tool can be applied to either one crutch or two, although we obviate an item in the form of two crutches: PSGD, as the scapular girdle remains static when advancing with both crutches in parallel.

The scale is applied in 2 minutes, maximum.

### Measurement of intra-rater and inter-rater reliability

Two raters (A and B) blinded one to the other and with experience in assisted gait with crutches participated in the study. They had previously been trained and each of the 10 items to be assessed had been fully explained. The raters first performed a pilot study on extra individuals in the sample. Afterwards, they assessed the study sample.

Both raters in separate rooms visualized the recordings for all participants on two occasions (A1, A2, B1 and B2). The second occasion took place a week later. Data that were considered as influential factors in the performance of assisted gait with crutches were also registered on the recording sheet. These included: laterality (manual, foot and ocular), previous experience with assisted gait and normal sports activity involving a coordinative skills development, amongst others.

### Data analysis

Shapiro-Wilks test was carried out before the main statistical analysis to analyze the normal distribution of study variables.

#### Scale construction and validity

We calculated a **correlation matrix** which expresses the relationship among all the elements evaluated in the subjects, in order to check the redundancy of items. Correlations under 0.30 indicate a low relation between the items. On the contrary, two items would be considered as redundant if their Spearman correlation coefficient exceeded 0.80[9].

The **ceiling and floor effects** were checked by calculating the percentage of subjects with very low or very high scores defined on the base of the score range. We considered these were present when >10% of subjects achieved the highest or the lowest scores[32].

The **criterion-related validity** was determined using Spearman correlation coefficient as the data did not follow a normal distribution in any case. The magnitude of correlation was classified as follows: 0.26–0.49, low; 0.50–0.69, moderate; ≥0.7, strong[33,34]. This validity was assessed by correlation between the CHAGS group of items that refers to the parameters of normal gait, independent to the use of crutches and Viel Coding[28]. The items that composed Viel Coding were: attitude during gait that measures fluency; gait variability which evaluates rhythm; loss of serious balance intended for the pathological lack of coordination and balance on some neurological patients; decision about heel contact, associated with an equine or steppage gait; hip extension, related to the neurological patients who maintain a hip flexion during the step; synchrony between the upper and lower limb, which assess pelvic and scapular girdle dissociation; distance between feet on the floor that evaluates step length symmetry; and duration of double support, which studies the lack of decision to start a step[35].

#### Reliability of the scale

**Internal consistency** was assessed with the Cronbach’s alpha coefficient. The score correlation of each item with the score from the rest of the Scale, that is, with the exception of this item, was also analyzed. Regarding this coefficient, values equal or higher than 0.70 were considered adequate[36]. Therefore, the item-total correlation of the elements was contemplated, expecting correlations equal to or higher than 0.30.

We conducted a **descriptive analysis** of the results obtained in different measurements carried out on the Scale. This was performed for unilateral assisted gait (dominant and non-dominant arm were distinguished) and bilateral, each of them evaluated by two raters (A and B) at two different moments (A1, A2, B1 and B2).

An item-by-item analysis was performed for **intra and inter-rater reliability** (weighted kappa coefficient). Score criteria described by Landis and Koch[37] were used. These criteria consider kappa as “almost perfect” >0.80, “substantial” from 0.61 to 0.80, “moderate” 0.41–0.60, “fair” 0.21–0.40, “slight” 0.00–0.20 and “poor” below 0.00.

The **minimal detectable change** (MDC) described by Weir[38] and the MDC% were calculated[39].

$$\text{MDC}=1.96\cdot \sqrt{2}\cdot 1.96$$

The Intra-class Correlation Coefficient (ICC) determined the **relative reliability** by using a two-factor model with mixed effects (ICC A) with absolute agreement, and a one-factor model with random effects (ICC B). When ICC B is equal to ICC A, it is because no systematic error is present[28]. Besides, we obtained the standard error of measurement (SEM) and the SEM %, according to Weir[38].

$$\text{SEM}=\text{SD}\;\sqrt{1-\text{ICC}}$$

All data analyses were conducted using SPSS 18.0 and MedCalc software. The statistical tests were performed considering the confidence interval of 95% (p<0.05).

## Results

### Scale construction and validity

All the correlations between the items showed ideal values, between 0.30 and 0.79. Nevertheless, some items showed correlations below (25 out of 760) or above said interval (22 out of 760).

The floor and ceiling effects were not present, although in the unilateral assisted gait between 50 and 63.3% of the participants obtained the same or higher scores than 34 (A1:63.3%; A2:50%; B1:56.6%; B2:63.2%) in the observations; and between 13.3 and 16.7% of the subjects obtained scores of 39 or 40 (A1:16,7%; A2:16,7%; B1:16,6%; B2:13,3%). Regarding bilateral assisted gait, between 46.7 and 56.6% of the participants obtained the same or higher scores than 33 (A1:53.3%; A2:56.6%; B1:46.7%; B2:53.4%) and between 30 and 36.7% of the subjects obtained scores of 35 or 36 (A1:33.4%; A2:30%; B1:36.7%; B2:33.4%). Between 3.3 and 10% of the individuals obtained a score of 40 (maximum score).

CHAGS correlated highly and negatively with Viel Coding[28]. Spearman’s minimum coefficient correlation in the unilateral assisted gait was -0.83 (A1:-0.91; A2:-0.85; B1:-0.89; B2:-0.83); and it was -0.67 (A1:-0.78; A2:-0.67; B1:-0.78; B2:-0.71) in the bilateral assisted gait.

### Reliability of the scale

Cronbach’s alpha oscillated between 0.90 and 0.91 in the modality of one crutch (A1:0.91; A2:0.91; B1:0.90; B2:0.91); and between 0.87 and 0.91 in the modality of two crutches (A: 0.91–0.91; B: 0.89–0.87). In all cases, the values of this coefficient were above 0.70. These results showed good internal consistency. In addition, all item-total correlations were above 0.30, indicating a good discrimination capacity of items within the assessed construct.

The results of the descriptive analysis for the subjects in each of the evaluations are shown in Table 1.

**Table 1:** Descriptive analysis of the results obtained in each measurement carried out on CHAGS scale.

CHAGS MEASUREMENT				MEAN OF EACH PAIR OFMEASUREMENTS				DIFFERENCE OF EACH PAIR OFMEASUREMENTS			
	MEAN	SD	MAX/MIN		MEAN	SD	MAX/MIN		MEAN	SD	MAX/MIN
**DH CHAGS A1**	35.18	5.15	40/25	**DH A1 A2**	34.79	5.34	40/24	**DH A1 A2**	0.76	1.03	3/-1
**DH CHAGS A2**	34.41	5.58	40/23	**DH B1 B2**	34.44	5.61	40/21.5	**DH B1 B2**	0.18	1.55	3/-3
**DH CHAGS B1**	34.53	5.62	40/23	**DH A1 B1**	34.85	5.36	40/24	**DH A1 B1**	0.65	1.17	3/-1
**DH CHAGS B2**	34.35	5.71	40/20	**DH A1 B2**	34.85	5.36	40/24	**DH A1 B2**	0.82	1.67	6/-2
**NH CHAGS A1**	30.41	6.77	38/14	**DH A2 B1**	34.47	5.58	40/23	**DH A2 B1**	0.12	0.99	2/-1
**NH CHAGS A2**	29.92	6.79	38/14	**DH A2 B2**	34.38	5.58	40/22.5	**DH A2 B2**	0.59	1.75	5/-3
**NH CHAGS B1**	30.00	6.78	38/15	**NH A1 A2**	30.19	6.77	38/14	**NH A1 A2**	0.54	0.78	2/-1
**NH CHAGS B2**	30.69	7.25	38/14	**NH B1 B2**	30.35	7.00	38/14.5	**NH B1 B2**	-0.69	0.95	1/-2
**BH CHAGS A1**	30.53	5.75	36/14	**NH A1 B1**	30.23	6.74	38/14.5	**NH A1 B1**	0.46	1.33	3/-2
**BH CHAGS A2**	30.63	5.68	36/15	**NH A1 B2**	30.23	6.74	38/14.5	**NH A1 B2**	-0.23	1.24	2/-3
**BH CHAGS B1**	30.67	5.47	36/16	**NH A2 B1**	29.96	6.77	38/14.5	**NH A2 B1**	-0.08	0.86	2/-1
**BH CHAGS B2**	30.83	517	36/17	**NH A2 B2**	30.31	7.00	38/14	**NH A2 B2**	-0.77	1.09	1/-3
				**BH A1 A2**	30.58	5.70	36/14.5	**BH A1 A2**	-0.10	0.76	1/-2
				**BH B1 B2**	30.75	5.30	36/16.5	**BH B1 B2**	-0.17	1.02	2/-3
				**BH A1 B1**	30.60	5.59	36/15	**BH A1 B1**	-0.13	0.86	1/-2
				**BH A1 B2**	30.60	5.59	36/15	**BH A1 B2**	-0.30	1.24	3/-3
				**BH A2 B1**	30.65	5.55	36/15.5	**BH A2 B1**	-0.03	1.03	3/-2
				**BH A2 B2**	30.73	5.40	36/16	**BH A2 B2**	-0.20	1.24	3/-3

Abbreviations: DH, dominant hand; NH, non-dominant hand; BH, both hands; A1, first assessment of rater A; A2, second assessment of rater A; B1, first assessment of rater B; B2, second assessment of rater B. The table includes the two measurements made with DH, NH and BH of raters A and B, the means of each pair of measurements compared and the differences between each pair of measurements.

As seen in Table 2, the weighted kappa had values ranging from 0.46 to 1.00. Most of the weighted kappa (60 out of 114) had values >0.80.

**Table 2:** Intra and inter rater reliability: weighted kappa coefficients for the CHAGS items in unilateral and bilateral conditions.

ASSISTED GAIT	RELIABILITY	INTRA-RATER		INTER-RATER			
	CONTRAST	A1-A2	B1-B2	A1-B1	A1-B2	A2-B1	A2-B2
**UNILATERAL**	Pelvic dissociation	0.85	0.81	0.92	0.79	0.82	0.85
	Deviation COG	0.86	0.73	0.92	0.81	0.78	0.82
	Crutch inclination	0.84	0.65	0.72	0.46	0.70	0.47
	Step rhythm	0.94	0.81	0.81	1.00	0.76	0.94
	Symmetry step length	1.00	0.89	0.71	0.74	0.71	0.74
	Cross support	0.84	0.78	0.77	0.60	0.70	0.60
	Simultaneous support	0.83	0.83	0.83	0.85	0.76	0.82
	Forearm off	0.90	0.84	0.63	0.63	0.74	0.92
	Facing forward	0.75	0.72	0.60	0.58	0.64	0.56
	Fluency	0.91	0.86	0.84	0.84	0.77	0.77
**BILATERAL**	Deviation COG	0.65	0.65	0.46	0.65	0.65	1.00
	Crutch inclination	0.96	0.92	0.92	0.84	0.88	0.81
	Step rhythm	1.00	0.83	0.92	0.92	0.92	0.92
	Symmetry step length	0.81	0.76	0.87	0.65	0.79	0.71
	Cross support	0.91	0.77	0.82	0.73	0.73	0.75
	Simultaneous support	0.97	0.97	0.92	0.94	0.89	0.92
	Forearm off	0.93	0.81	0.56	0.67	0.66	0.61
	Facing forward	0.86	0.81	0.75	0.67	0.78	0.70
	Fluency	1.00	0.91	0.83	0.76	0.83	0.76

Abbreviations: A1, first assessment of rater A; A2, second assessment of rater A; B1, first assessment of rater B; B2, second assessment of rater B.

We found a high level of reliability with all ICC close to 1. ICC A and B, SEM and MDC, calculated with confidence intervals (CI) at 95% are shown in Table 3 (ICC A and B were always the equal number). The values of SEM for each observation and evaluator, which oscillated between 0.03 and 0.24, are shown in Table 4.

**Table 3:** ICC (95% CI), SEM and MDC (95% CI) of the compared measurements.

ASSISTED GAIT	RELIABILITY	INTRA-RATER		INTER-RATER			
	Compared Measurements	A1-A2	B1-B2	A1-B1	A1-B2	A2-B1	A2-B2
**Unilateral**	ICC (95% CI)	**0.99**	**0.98**	**0.98**	**0.97**	**0.99**	**0.97**
	SEM	0.63	0.91	0.90	1.10	0.64	1.12
	MDC (95% CI)	1.75	2.54	2.48	3.04	1.78	3.10
**Bilateral**	ICC (95% CI)	**0.99**	**0.98**	**0.98**	**0.97**	**0.98**	**0.97**
	SEM	0.57	0.75	0.79	0.97	0.78	0.93
	MDC (95% CI)	1.58	2.07	2.19	2.68	2.17	2.59

Abbreviations: ICC, intra-class correlation coefficient; CI, confidence interval; SEM, standard error of measurement; MDC, minimal detectable change; A1, first assessment of rater A; A2, second assessment of rater A; B1, first assessment of rater B; B2, second assessment of rater B.

**Table 4:** SEM for each evaluator and observation.

ASSISTED GAIT	RELIABILITY	A1		A2		B1		B2	
	CONTRAST	MEAN	SEM	MEAN	SEM	MEAN	SEM	MEAN	SEM
**UNILATERAL**	Pelvic dissociation	2.50	0.22	2.30	0.22	2.53	0.21	2.43	0.21
	Deviation COG	3.30	0.15	3.17	0.17	3.37	0.16	3.27	0.15
	Crutch inclination	3.57	0.10	3.47	0.12	3.53	0.12	3.57	0.14
	Step rhythm	3.70	0.12	3.67	0.12	3.67	0.13	3.70	0.12
	Symmetry step length	3.63	0.14	3.63	0.14	3.67	0.14	3.60	0.16
	Cross support	3.37	0.15	3.37	0.16	3.23	0.16	3.43	0.16
	Simultaneous support	2.67	0.23	2.70	0.23	2.63	0.24	2.73	0.22
	Forearm off	3.83	0.09	3.80	0.09	3.77	0.11	3.77	0.12
	Facing forward	2.77	0.16	2.60	0.16	2.43	0.18	2.53	0.19
	Fluency	3.80	0.11	3.77	0.11	3.73	0.13	3.73	0.13
**BILATERAL**	Deviation COG	3.93	0.05	3.97	0.03	3.93	0.05	3.97	0.03
	Crutch inclination	3.13	0.16	3.10	0.16	3.20	0.15	3.20	0.15
	Step rhythm	3.73	0.11	3.73	0.11	3.77	0.10	3.77	0.10
	Symmetry step length	3.67	0.13	3.70	0.14	3.73	0.11	3.77	0.09
	Cross support	3.00	0.21	3.03	0.20	2.77	0.23	2.93	0.23
	Simultaneous support	3.00	0.21	2.97	0.21	2.97	0.22	3.00	0.21
	Forearm off	3.70	0.11	3.67	0.12	3.77	0.11	3.83	0.08
	Facing forward	2.63	0.21	2.73	0.20	2.73	0.20	2.63	0.21
	Fluency	3.73	0.11	3.73	0.11	3.80	0.09	3.77	0.10

Abbreviations: SEM, standard error of measurement; A1, first assessment of rater A; A2, second assessment of rater A; B1, first assessment of rater B; B2, second assessment of rater B.

## Discussion

Throughout time, different authors such as Toro[40] and Chamorro[17] have criticized a systematic lack of gait assessment tools for patients with musculoskeletal injuries. The study, in the same line of criticism, tried to record and reduce, by means of creating the CHAGS, the need to systematize assisted gait assessment in lesions that require the use of crutches. This paper shows the designed Scale is a valid and reliable tool for assessing assisted gait with crutches in subjects with sprained ankles.

Regarding the statistical results obtained on reliability and validation of the Scale, we realized that the items were not redundant, although some of them had correlations exceeding 0.80[9]. However, no systematic patterns of high or low frequency were observed, so that the results did not suggest removing any item from the Scale. The items that had correlations above 0.80 were: “step rhythm” and “symmetry of step length”; “cross support” and “simultaneous support of foot and crutch” and “step rhythm” and “fluency”.

In regard to the first group, “step rhythm” and “symmetry of step length”, when the gait speed is constant and the step length varies with respect to the other step, the rhythm changes as a result of the difference between the right and the left step periods[41].

With respect to the second group, “cross support” and “simultaneous support of foot and crutch”, subjects with good coordination which focus especially on crutch support perform fast uniform linear motions rhythmically, coplanar with the same angle between the upper limb carrying the crutch and the affected limb that is taking the step. These three characteristics correspond to a motor plan easier to calculate by our cerebellum, thus favoring the fluidity and economy of movement[42]. Therefore, the degrees of hip flexion on taking the step, and those of shoulder flexion on advancing the crutch (arm extension) are similar to free cadence and, consequently, the support coincides with the side of the foot.

Finally, the third group was composed of “step rhythm” and “fluency”. There are scales, such as “Modified Gait Abnormality Rating Scale”[43] that assess both parameters in the same item. CHAGS authors decided to evaluate them separately so as to find differences between them. Fluidity was associated with movement automation and absence of rigidity so that a subject can walk at a correct rhythm but in block and without swinging their arms.

Despite the absence of the ceiling effect, especially high scores were obtained which was to be expected as most of the patients came from the field of sports and possessed a high level of coordination. Moreover, except for a few subjects, these had been patients at Physiotherapy Centers for several weeks where they performed an explicit learning process and functional gait recovery. These coordinative skills and level of learning allowed patients to walk correctly, independently of their pathology, while performing assisted gait with forearm crutches, a dual-task where the crutch is distracting subject gait[19]. The floor effect was not present due to the absence of low scores. So, the minimum score recorded reached 14 points in both unilateral and bilateral assisted gait. The reason for this was the presence of subjects who had been performing assisted gait with crutches for a short time because of the short evolution of their injury and a lack of coordination which made the learning of using crutches difficult.

In relation to the criterion-related validity, this study showed that CHAGS has strong correlations when compared with Viel Coding[28]. 87.5% of the measurements confirmed that a high correlation exists and only one was below what is considered strong. Therefore we justify this measurement in the sample size, which is not high. Only the group of items that constitutes normal gait parameters, regardless of the use of a crutch, were correlated: “PSGD”, “deviation from the COG”, “step rhythm”, “symmetry of step length”, “look forward” and “fluency”. The correlation between both scales is negative as Viel Coding[28] has low scores in the absence of dysfunction, as opposed to CHAGS.

With regard to the descriptive analysis, and bearing in mind the unilateral gait is contra-lateral, i.e. the crutch is on the opposite upper limb to the lower limb injury, results were as expected by the authors. Participants who held the crutch in their dominant hand scored better than those who held it in their non-dominant hand. These findings corroborate that the laterality, and therefore, coordination, greatly influences the execution of the assisted gait and, consequently, the score of the CHAGS. Moreover, the results were slightly higher in the modality of two crutches than in the non-dominant unilateral gait. Obtaining the best results in CHAGS is influenced, therefore, by the dominance of the upper limb that holds the crutch, and even expands this idea, by the coordinative abilities of the subject that determines the presence of a good crutch control.

All the results obtained in the analysis of reliability assessment of CHAGS were favorable. Regarding the weighted kappa indexes, most of the results were “almost perfect” while none of them was below what is considered moderate by Landis and Koch.

Although this scale can be used in the assessment of unilateral or bilateral assisted gait, in the latter, the item PSGD is not included. This is due to the use of two crutches in a two-tempo gait which forces the scapular girdle not to rotate, in turn eliminating the alternating arm swing. Initially, the authors decided to remove PSGD as well as “displacement of the COG” as a consequence of the supposed symmetrical support on both sides. However, during the development of the Scale, we found that some patients altered their COG because of the asymmetry of their loads and the difference in inclination between both crutches. For this reason, we decided to consider “displacement of the COG” in the CHAGS variable intended for bilateral assisted gait.

Regarding the clinical application of the Scale, a training period is required for the observers (3 to 5 patients), which is facilitated for physical therapists that usually work on gait re-education. The training period is brief due to CHAGS ease-of-use. Each parameter is observed independently, thus it is an analytic evaluation. In this sense, to focus the attention on a specific parameter allows an efficient assessment. However, a general view is always needed. Moreover, all items have the same scoring model, which also facilitates the assessment. This study validates CHAGS scale for subjects who suffer from sprained ankles, although the tool is intended for the assessment of all patients that precise the use of crutches due to a musculoskeletal injury.

Besides being a tool for initial, ongoing and final assessment, the CHAGS functional scale is a feedback system that can be used to identify specific parameters of assisted gait, by means of each item, that requires a specific task during each patient’s treatment. That is why the Scale is interpreted item-by-item and not globally as indicated in the “Development of CHAGS” section. Therefore, CHAGS acquires a relevant role in the clinical setting, and will facilitate the determination of management protocols based on scientific evidence[7].

Finally we believe that the design and validation of the CHAGS besides the reliability study, provides a useful new tool for analysis and functional assessment of assisted gait with crutches. It is easy to use and requires no spatial, temporal and economic resources, thus making it a feasible assessment procedure in daily clinical practice while being objective, rigorous and efficient at the same time[44].

### Study limitations

This study presented a limitation regarding the experience of observers. As this is the first research of a new assessment tool, the assessors had a limited experience in its use. The development of more studies on CHAGS will solve this limitation.

The results shown in this article can only be considered for people walking with crutches and having sprained ankles, and not all patients walking with crutches, whatever their pathology. A prospective study on healthy subjects could demonstrate that assisted gait disturbances depend on the use of crutches and are independent of the pathology.

Although CHAGS scale analyzes gait from the point of view of a step as a basic unit of gait and measures, the absence of kinematic and balance parameters can be a study limitation.

## Conclusion

The CHAGS rating scale, a new means for evaluating aided gait with forearm crutches to partially relieve an affected member due to a musculoskeletal injury, has been designed and found to be valid and reliable in subjects with sprained ankles. This scale should be applied to other types of subjects prospectively. A good internal consistency and high correlation with Viel Coding were obtained by CHAGS scale.

The innovative tool constitutes a feasible measurement method in daily clinical settings while being objective, rigorous and efficient at the same time.

## Supporting Information

S1 Appendix Chamorro Assisted Gait Scale (CHAGS). ^a^Functional Rating Scale of assisted gait with partial discharge by means of Canadian crutches. ^#^Items unique to gait mode with a single crutch. ^$^Items valid for gait with one or two crutches.
(DOCX)
