# Supplementary material for: Reliability and Validity Study of the Chamorro Assisted Gait Scale for People with Sprained Ankles, Walking with Forearm Crutches
Source: PLoS One. 2016 May 11;11(5):e0155225. doi: 10.1371/journal.pone.0155225 (PMC4864073; doi:10.1371/journal.pone.0155225)
Supplement: S1 Appendix — aFunctional Rating Scale of assisted gait with partial discharge by means of Canadian crutches. #Items unique to gait mode with a single crutch. $Items valid for gait with one or two crutches. (DOCX) [file pone.0155225.s001.docx]

**Chamorro Assisted Gait Scale (CHAGS)**

1. Pelvic and scapular girdle dissociation^#^
0.- No dissociation between both girdles is carried out. The patient does not swing their arm or their contra-lateral arm at the same time as the crutch.
1.- They do not perform any dissociation between both girdles. They swing their arms without fluency.
2.- They perform dissociation between both girdles discreetly or with slight fluency. They swing their arms without fluency.
3.- They carry out dissociation between pelvic and scapular girdle with slight fluency. The contra-lateral arm is slightly loose.
4.- They perform dissociation between pelvic and scapular girdle with fluency. Contra-lateral arm is loose.

2. Deviation of Center of Gravity (hereinafter COG)^$^
0.- The COG is on the crutch.
1.- The COG is between the healthy foot and cane.
2.- The COG is on the healthy foot.
3.- The COG is between both feet.
4.- The COG is centered between both feet.

3. Crutch inclination^$^
0.- The crutch arm is maintained separate from the body and said crutch is not aligned with the arm, but is angled outwards.
1.- The crutch arm is kept separate from the body with the aligned cane.
2.- The arm is slightly separated from the body with the aligned cane.
3.- The arm supports the aligned crutch which is maintained throughout the body, but not always.
4.- The arm supports the aligned crutch which is maintained throughout the body.

4. Step rhythm^$^
0.- The tempo is never performed correctly, i.e., each step has a different duration to the contra-lateral.
1.- The tempo is hardly ever performed correctly.
2.- The tempo is sometimes performed correctly.
3.- The tempo is nearly always performed correctly.
4.- The tempo is always performed correctly, i.e. all steps are carried out in the same time.

5. Step length symmetry^$^ (take note whether the step of the affected member is longer or shorter)

0.- The right step and the left step never have the same length.

1.- The right step and the left step hardly never have the same length.

2.- The right step and the left step sometimes have the same length.

3.- The right step and the left step nearly always have the same length.

4.- The right step and the left step always have the same length.

6. Cross support^$^
0.- Affected foot never laterally supports the crutch.
1.- Affected foot hardly ever laterally supports the crutch.
2.- Affected foot sometimes laterally supports the crutch.
3.- Affected foot almost always laterally supports the crutch.
4.- Affected foot always laterally supports the crutch.

7. Simultaneous support of foot and crutch^$^
0.- The affected foot support is never carried out at the same time or just after the crutch or crutches support.
1.- The affected foot support is hardly ever carried out with a crutch or crutches.
2.- Affected foot support is sometimes performed with a crutch or crutches.
3.- Affected foot support is almost always performed with a crutch or crutches.
4.- Affected foot support is always carried out with a crutch or crutches.

8. Forearm off^$^
0.- The forearm is always separate to the crutch.
1.- The forearm is almost always separate to the crutch.
2.- The forearm is sometimes separate to the crutch.
3.- The forearm is hardly ever separate to the crutch.
4.- The forearm is never separate to the crutch.

9. Facing forward^$^

0.- They never face forward.

1.- They hardly ever face forward.

2.- They sometimes face forward.

3.- They almost always face forward.

4.- They always face forward.

10. Fluency^$^
0.- There is no fluidity in the gait, stopping at some point on the way and / or rectifying affected foot support, crutch or crutches.
1.- There is no fluidity in the gait due to indecision when supporting the crutch or taking a step, but without completely stopping or rectifying the supports.
2.- Performs assisted gait with slight fluency, i.e. with certain determination and some hesitation.
3.- Performs assisted gait decisively and without hesitation, but requires constant concentration.
4.- Performs the assisted gait automatically, decisively and without hesitation.

^a^Functional Rating Scale of assisted gait with partial discharge by means of Canadian crutches. ^#^Items unique to gait mode with a single crutch. ^$^Items valid for gait with one or two crutches.
